# Prescriptive ‘selves’ and self-illness ambiguity

**DOI:** 10.1007/s11229-025-05147-8

**Published:** 2025-07-17

**Authors:** Jodie Louise Russell

**Affiliations:** Institute for Mental Health, University of Birmingham, Birmingham, UK

**Keywords:** Self-illness ambiguity, Alienation, Normativity, Mental disorder, Mind-shaping

## Abstract

Recent work on the phenomenon of self-illness ambiguity has sought to not only understand how tensions arise between one’s experience of self and one’s disorder experiences, but also how best to resolve said ambiguities to relieve the suffering of the person in question (Sadler, Psychiatry: Interpersonal and Biological Processes, 70(2), 113–129, 2007; Dings & Glas, Philosophy, Psychiatry, & Psychology, 27(4), 333–347, 2020; Dings & de Bruin, American Journal of Bioethics, 22(6), 58–60, 2022; Jeppsson, Philosophical Explorations, 25(3), 294–313, 2022). While the involvement of other people has been emphasised as important in regulating the self and thus will play a part in self-illness ambiguity, the impact of this social dimension has not been sufficiently explored. The goal of this paper is to provide an account of how social norms may be implicated in the enactment of the ‘self’ and experiences of self-illness ambiguity. To do this, I will provide a plausible account of what it means to have a coherent or understandable self, drawing on the mind-shaping view of social cognition (McGeer, Folk Psychology Re-Assessed, 137–156, 2007; Mameli, Biology & Philosophy, 16(5), 595–626, 2001; Zawidzki, Philosophical Explorations, 11(3), 193–210, 2008; Zawidzki, Mindshaping: A new framework for understanding human social cognition. Cambridge, MA: MIT Press, 2013; Zawidzki, Mindshaping and self-interpretation. In J. Kiverstein (Ed.), The Routledge Handbook of Philosophy of the Social Mind, 495–513. London & New York: Routledge, 2016). Mind-shaping suggests that we are successful in social coordination tasks because we are able to negotiate and follow shared norms that facilitate understanding. These norms indicate and shape what kinds of people we understand ourselves to be, as well as how others understand us, which we might refer to as the ‘self’. Given that disorder experiences can be transformative, fundamentally changing how the world is meaningful for someone, we may therefore expect that disorder experiences can transform norms of the self that may undermine seamless social coordination. Following this, I argue that individuals with self-illness ambiguity face unique challenges when it comes to navigating social problems that other individuals with disorder experiences may not face. This is because, as I argue, some discourses around mental disorder are deemed more or less *valuable* strategies for conceptualising the relationship between self and disorder. Since self-illness ambiguity doesn’t ‘fit’ into these discourses, individuals with self-illness ambiguity may feel isolated not just from their wider community but also from mental disorder communities themselves. I suggest, then, that individuals with self-illness ambiguity might experience an acute form of alienation that is yet to be discussed in the literature.

## Introduction

Self-ambiguity occurs when it is unclear to an individual whether an experience or behaviour is part of their sense of ‘self’ or whether it is due to some other factor (Dings and De Bruin, 2022). For example, it may be unclear whether I am personally upset with my friend for being late (self-attribution) or whether I am simply hungry (bodily attribution), resulting in a kind of self-body ambiguity. Individuals with mental disorders are especially prone to experiences of self-ambiguity, or, more specifically, experiences of self-*illness* ambiguity where the tension lies between one’s ‘self’ and a mental disorder experience. Sadler (2004) compares physical illness and mental disorder to make this clear. In physical illness, such as when someone is diagnosed with cancer, the illness is often referred to as an external imposition on the individual herself (to use Sadler’s example, “She was struck down by cancer”). This isn’t a mere metaphor on Sadler’s view as individuals *experience* their sense of self as unambiguously separate from their physical illness^1^.

In mental disorder, however, this experience of difference is blurred. In Sadler’s view, mental disorder saturates or transforms a person’s relationship to the world, such as when someone’s distressing delusions make the world feel threatening and the person in question feels paranoid. Someone may also value their disorder experiences or wish to preserve them because they have personal meaning (Sadler, 2004) such as, for example, when someone finds comfort and reassurance in the voices they hear. In such cases, the experience of mental disorder can become intertwined with other experiences one might typically attribute to one’s sense of self (e.g. their values, desires, and relationships to the world). It then becomes tricky to discern whether an experience or behaviour is as a result of one’s self or one’s illness. Participant 23 in Hope et al.’s (2011) study describes such an experience: “Am I being anorexia because I’m not having a spread, or is that me being perfectly capable of making my own decision, about what I do and don’t like?” (p.25).

Unfortunately, it is because of this blurred boundary that individuals with mental disorder may face blame, stigma and vilification. This is because the behaviour of those with mental disorder is often perceived as “incomprehensible, annoying, or bizarre” (Sadler, 2004, p.1811). This suggests to me that there are certain social expectations when it comes to mental disorder and the self, such that the blurring of boundaries between the two is not considered understandable or acceptable. My intention for this paper is to explicate the relationship between the social norms of acceptability, social understanding and mental disorder to consider the consequences for those with disorder ambiguities. I propose that there is a wide-spread, societal expectation to present a coherent ‘self’, i.e. a kind of person that other people can understand. I argue that community-wide prescriptions to be understandable to others poses unique problems for individuals with disorder ambiguities which are yet to be discussed in the literature.

To make this case, I will first provide background on the nature of self-illness ambiguity and the particular problems it can pose for individuals who experience it (§2). I will then outline the mind-shaping view of social cognition, which describes social understanding as a process of adjudicating between and conforming to sets of norms, and derive an embodied account of the self from it (§3). I then argue that mental disorder experiences can become implicated in the social processes of understanding, especially when they transform our habits and meaning structures. This can make it hard to remain understandable by others, which is why mental disorder comes into tension with the self in self-illness ambiguity (§4).

With a mind-shaping account of self-illness ambiguity in hand, I then make the case that wider social structures are implicated in prescribing self and disorder discourses that can be incommensurable with experiences of self-illness ambiguity (§5). This leads to unique kinds of alienation for individuals with self-illness ambiguity when their experience doesn’t fit useful disorder discourses. This highlights an additional barrier to being understood faced by those with self-illness ambiguities and may constitute a hermeneutic injustice (see Fricker, 2007).

I wish to make a few points of clarification before I proceed. The term ‘illness’ in self-illness ambiguity is intended to specifically relate to experiences of mental disorder (although much of the discussion below, especially in terms of the transformative nature of illness may apply to somatic illness as well). More specifically, I am talking about disorder experiences that are distinctly transformative, i.e. the perspective of the person has changed, either radically or subtly (in other words, cases where someone has become depressed, or started to experience delusions, where this was not the case before). It might not be clear when the onset of the transformation began, but there has certainly been a change in the person’s experience that we might attribute to mental disorder. It may be the case that there are disorder experiences someone has had their whole life, or disorder experiences that are intermittent or transient, where ambiguities also occur. However, I focus on transformational mental disorder in order to simply motivate the argument that norms are implicated in mental disorder understanding. I am not intending to delineate what counts as mental disorder and so I leave it open to discussion how self-illness ambiguities come about in other disorder experiences.

Moreover, my discussion of illness below is not intended to capture experiences of cognitive difference, i.e. neurodivergence, although there might be similar ambiguities at play. There may also be many points in §5 that resonate with experiences of neurodivergence regarding the necessity to ‘fit in’ with acceptable ways of talking about non-typical experiences, and there may be instances of neurodivergent ambiguities that are relevant and worthy of discussion within this context, but this isn’t within my scope here.

My use of the term ‘illness’ is also not intended to pathologize self-illness ambiguity nor mental disorder experience. However, because of its connotations, Mad-identified individuals who have had the transformative experiences I describe may legitimately feel uncomfortable. I use the term “self-illness ambiguity” only to maintain an explicit conceptual link to the literature, but I don’t think it is necessary to refer to mental disorder as illness when talking about ambiguities. I would therefore encourage the reader to insert their preferred term with regards to ambiguities when reading this paper, be that mental illness, mental disorder, madness, altered states of consciousness or something else (e.g. “self-madness ambiguity” or “self-disorder ambiguity”).

## Background: Self-illness ambiguity

Dings and de Bruin (2022) understand self-illness ambiguity to refer to the experience of vagueness or uncertainty felt by individuals with mental disorder over whether a particular emotion, behaviour or thought can be said to be as a result of who they are as a person (their self) or as a result of their mental disorder. For example, a person may feel despondent and guilty for not doing enough work in a day. Self-illness ambiguity may arise if this person struggles to disambiguate whether their feelings have arisen due to some feature of them as a person, e.g. they find the work challenging or unenjoyable but feel they should have been able to overcome that, or whether their feelings are a symptom of depression. Dings and de Bruin note further that self-illness ambiguity is part of a much wider experience of ‘self-ambiguity’, which may include ambiguities between experiences of the self and other factors (like bodily feelings). We may, for example, lose our temper at a friend for forgetting plans for dinner and subsequently question whether our outburst was because we are a short-tempered person or whether we were simply hungry at the time. We may feel, in this case, that we were not completely in control of our actions (the outburst) but, at the same time, we also weren’t forced or coerced into taking that action (Dings & Glas, 2020).

While the concept of self-illness ambiguity is relatively new (see Sadler, 2004, 2007; Dings and De Bruin, 2023), there has been much work attempting to explicate the phenomenon. One way this has been achieved is through a broader understanding of the ‘self’. Sadler (2007), for instance, refers to the self as ‘multidimensional’ and ‘personal’, which incorporates many factors and may be instantiated over different levels. On his account the self is an agential identity with a history and goals, a purpose, and a view on the world that separates itself from others. The personal self is also turned towards future pursuits while being informed by the past (Sadler, 2007). Importantly, Sadler also notes that the personal self is experienced as something that is *owned* or that belongs *to me*, not as something theoretical or abstract. When I question whether *I* am short tempered, I am asking whether my anger at my friend’s forgetfulness is part of the self that I experience *as me*. This ‘me’ is multimodal in the sense that what I feel to be ‘mine’, or what belongs to my ‘self’, spans different ontological levels or domains (e.g. bodily, psychological and, importantly, social levels).

Bortolan (2022) compliments this multimodal picture. She argues that self-illness ambiguity may arise through the way we affectively navigate the environment, i.e. through our emotions. Maiese (2022) additionally emphasises the role of sensorimotor capacities through the relevance of affordances to the sense of self, where self-ambiguity may also arise when a sense of ‘mine-ness’ is compromised. This may happen when a particular action-possibility can’t be fully integrated into the story of what actions are possible for a person like me. Dings and Glas (2020) also note how the process of regulating the self is an interpersonal process, and so ambiguity itself may be co-regulated by others. We see this in cases of self-illness ambiguity where individuals help ‘disambiguate’ illness-behaviours from self-behaviours; as participant 18 in Hope et al.’s (2011) study, describes, for example,

“[…] I’ll say something and my mum will turn around and say, “That’s anorexia,” and I’m like, “Ugh, what? That’s actually me,” and she’ll go, “No, you know, it’s anorexia.” So that in that respect, I get scared sometimes because I think, “oh my God, I don’t know who’s who!”” (p.25).

I will return to this particular point regarding the role other people play in the regulation of self in the sections below. It suffices to say here that the picture the literature on self-illness ambiguity presents is of an embodied, interpersonal, emotional and temporal experience of self. Mental disorder is also importantly embodied. We may feel, for example, our thoughts to be contained within our body or our disorder related behaviours to unfold from it. When we go about pursuing our life goals, then, it may not always be clear whether our experiences or behaviours are attributable to a mental disorder or our self, especially as they would seem to unfold from the same space (our bodies).

The literature is divided, however, on whether self-illness ambiguity is something that needs to be resolved, and if so, how it should be done^2^. Nevertheless, it is possible that ambiguities can cause a number of practical issues which may create barriers to achieving one’s personal goals (which may or may not include treatment). As Dings and de Haan (2022) note, how one relates to one’s mental disorder shapes how clinical decisions are made or may even determine whether clinical intervention is necessary at all. The relationship one has to one’s mental disorder is also something that changes over time, they argue, and therefore how one’s mental disorder is treated may also need to be adapted over time. It may therefore be pertinent to some clinical goals to be able to attribute an experience to *you* or *your disorder* in order to know what and how to treat it. Moreover, Sadler (2007) describes how many clinical structures – such as psychoeducational programmes, psychotherapy, and rehabilitation – are designed on the principle that patients should focus on tackling issues caused by their disorder in order to avoid creating internal conflict with themselves, and this is important for treatment to be successful.

Resolving self-illness ambiguity may also be relevant for helping individuals to overcome feelings of guilt, shame and blame for some thoughts and behaviours by being able to separate what is a result of one’s mental disorder from who one is (Jeppsson, 2022). Such ambiguities may also present existential problems when it’s unclear where one’s goals, intentions, feelings and inclinations even come from. This may make it additionally ambiguous as to what goals to pursue and how to even pursue them. For example, Benschop and Kalis (2025) describe that eating disorder behaviours themselves can demonstrate features of agency, such as flexibility, and can form part of one’s life project (which involves shaping one’s physical and social environment for the pursuit of eating-related goals). Individuals with eating disorders may experience existential problems in determining their life meaning if their ongoing projects are related to a mental disorder experience that they are trying to separate from their identity. In other words, people with eating disorders might find it difficult to determine ‘who they are’, because they are trying to understand themselves both in relation to and in separation of problematic eating-related life projects.

In the following sections, I want to consider an additional, underlying goal that complicates experiences of self-illness ambiguity. This is the goal to successfully coordinate with others in the social environment over time. As mentioned above, other people may form part of the structures that help delineate a ‘self’. By using the mind-shaping framework, I hope to show that there is an underlying need to present a self which others are able to understand and work with across different situations. However, certain ‘self’ identities are considered more valuable than others. Self-illness ambiguity discourses may be ones which are valued less precisely because ambiguities make it hard to maintain relationships of understanding and coordination. This makes people with these ambiguities uniquely alienated within the wider social domain. My account thus aims to capture the influence of sociality on self-illness ambiguity in a way not yet explored in the literature, and highlight additional problems individuals with ambiguities may face when pursuing personal goals.

## Mind-shaping and the self

The mind-shaping framework, I suggest, gives us the necessary tools to understand some of the social processes behind the development of the self as well as how mental disorder experiences can create tensions with the self in self-illness ambiguity. In what follows, I propose to use the mind-shaping framework to merely supplement or ‘flesh out’ the mechanics of the social realm that the multimodal self (as described above) will be partially instantiated by. In fully exploring the implications of social cognition on disorder experience, I argue that we can theorize new issues for those with self-illness ambiguity (see §5).

### Understanding others and ourselves

Mind-shaping is primarily a view about social cognition and aims to give an account of how we understand others (and ourselves). In contrast to traditional frameworks for social understanding whereby individuals attempt to glean meaning from behaviour (as in Theory Theory or Simulation Theory), regulative or mind-shaping approaches to social cognition claim that agents attempt to make themselves intelligible to others by showing their mutual participation in shared norms (e.g. McGeer, 2007; Mameli, 2001; Zawidzki, 2008, 2013, 2016). However, agents not only conform to norms but also guide the action of others in order to encourage them to conform to particular norms. The basis of this thought is that we have a stake in being intelligible to others (McGeer, 2007). It is in our best interest to make ourselves understandable to others because another’s interpretation of our behaviour can impact how successful we are in our goals. It’s useful, for instance, to have an agreed upon understanding of ‘predator’ between myself and my companions so that, when I spot one in the wild, we can appropriately coordinate our behaviour and flee together for safety.

One way to build a shared foundation for understandability is by trying to fit into some norm, or, alternatively, encouraging another to conform to your norm. Norms determine the available or ‘playable’ moves in social interactions, similar to the way that the rules of a boardgame constrain what counts as a proper move in the game (or even define the nature of the game itself; McGeer, 2015). For example, the interactions between a bookseller and customer are normatively regulated; the seller and customer must conform to shared rules in the interaction in order to make the purchase of a book successful. This is achieved through setting *expectations* about what one should do, or how one should act or behave (Mameli, 2001). The bookseller is expected to have books to hand, and a system by which the customer can legitimately buy them, e.g. a till or debit card system.

Expectations arise from *anticipations* of particular kinds of behaviour (and even thoughts and feelings) based on the type of person someone is categorised to be. Categorisation makes use of folk-psychological concepts, which are the psychological and conceptual toolset we, the ‘folk’, rely on to make sense of another’s behaviour. These tools can be characterised broadly, including anything from stereotypes, experiences of interacting with a particular individual in the past, social roles, tropes, even emotions or other socialising concepts like ‘love’ and ‘gratitude’ (McGeer, 2015). However, this list is plausibly inexhaustive (Andrews, 2015).

Importantly, these folk-psychological concepts contain norms to whom they should apply as well as expectations about how that individual should behave. In the example above, someone walking into a bookshop may categorize the person behind the counter as the bookseller (they conform to the norms and expectations we have of booksellers, e.g. they are standing in the right place in the shop, they are operating the till etc.). This categorization *itself* shapes the ongoing behaviour of the bookseller (i.e. because of the shared awareness of the norms in play between bookseller and customer, the bookseller is aware they are expected to, and may continue to, act in this role). It is in this way the bookseller and customer understand one another. Even when things go wrong other norms fill in the gaps to ‘explain away’ the interaction. If the customer’s card declines, but the bookseller hands over the book anyway, the customer might interpret this act as a ‘gift’ (or even a mistake) rather than a purchase. Our general social, and epistemic, goal of understanding one another is a constant negotiation of which norms and categorization apply to account for someone’s behaviour (what Andrews, 2015, terms the ‘folk-psychological spiral’).

According to Zawidzki (2016), *self*-understanding simply involves applying folk-psychological categories, and the norms/expectations they come with, to ourselves. These categories help us to determine and constrain meaningful action in the world. Heschel (1963) says, “Consciousness-of implies awareness of one’s special position in relation to other beings. Any *conception as to what I am going to do with myself presupposes my having an image of myself*” (p.8, emphasis added). For certain actions to appear as possibilities for me, I must have an idea of who I am first. This is something I draw from past folk-psychological categorizations that were applied to ‘me’ and/or the categories that apply in the current situation. In this way, self-understanding is an iterative process much like social understanding, where norms and expectations are constantly being negotiated for the task at hand.

It is therefore fruitful to have an understanding of who I am, i.e. what folk-psychological concepts can apply, because it allows me to carve up the environment in useful ways that *maintain* relationships of understanding with myself and others over time. Because the norms we conform to must be both flexible (to account for change, e.g. change in situation, body, expected circumstances) yet rigid (norms have to be something we can rely on, and that others rely on, to coordinate successfully), our self-understanding will also be both flexible and rigid. Our self-understanding is both constrained by the meaningful norms available (given the kind of person you are, or have shown to be, or what is simply available to do in the environment, with your kind of body) but it must also be adaptable to a range of different coordination tasks. Nevertheless, the way I understand myself and the way others understand me may fall into patterns insofar as I interact with the same/similar people and participate in the same/similar social milieus. In this way, it is possible that one’s self-understanding can calcify. This is not to say that we have no agency over our self-understanding, only to say that, following mind-shaping, social norms play a meaningful role in shaping, and even determining, the self.

### Dings’ embodied-narrative account of the self

It is important to highlight here the similarities between my view and Dings’ (2019, 2020) embodied-narrative account of the self, which informs his own account of self-illness ambiguity. On Dings’ view, our embodied subjectivity and narratively structured subjectivity overlap. That is to say, the experience of ourselves as a being with particular goals which reflect or relate to a narrative shapes what we do with our bodies, and what we do with our bodies also informs our sense of narrative. Put simply, self-narratives and embodiment mutually inform one another. In one direction, we deliberate over what aspects of our environment are relevant or important to us, which may constrain our actions moving forward, and thus self-narratives inform embodiment. Dings refers to this as “narrative self-programming”. In the other direction, the environment often affords for action and bodily intervention that may invite or prompt narrative deliberation (when, for example, our embodied habits run counter to narrative goals) or support narrative goals and meaning-making to give us the sense that our actions are ‘ours’.

The view I have adapted from mind-shaping shares many similarities with Dings’ embodied-narrative account insofar as the norms we are expected to conform to are also embodied. For instance, norms may come ‘bound’ with particular bodily phenomena. On an embodied and enactive account of emotions (see, for example, Colombetti, 2014), a case could be made that interoceptive experiences will be bound up with the enacting of our various ‘selves’. To be an angry person may be to act angry by gritting one’s teeth or tensing one’s shoulders, and this will come with the phenomenal experience of being angry.

As mentioned above, norms on the mind-shaping view can also become *habitual* forms of acting in the world when particular situations repeatedly prescribe particular ways of behaving. Gender may be seen as one example (see Young, 1980, as well as Butnor & MacKenzie, 2022). These norms then inform our perception of ourselves as a particular kind of person over time (but they may not be the be-all-and-end-all of “The Self”). This makes the norms of mind-shaping inherently embodied insofar as norms are constructed and reinforced through our continual enactment of them. In other words, I ‘act out’ my ‘self’ in a given situation. The individual is mind-shaped into being an angry person when a particular kind of situation calls for this particular ‘self’ (the angry person) to be enacted, but it becomes habitual through the reinforcing of the norm over time. A reputation as ‘the angry feminist’ may come about, for example, from the repeated stoking of colleagues with provocative comments which constantly call for the person in question to become angry. Being ‘the angry feminist’ may then, for better or for worse, become part of their identity, or, in other words, a self that they are expected to perform by others or themselves in order to maintain relationships of understanding.

Although I do not characterise the self in a narrative sense, my account is consistent with Dings’ in that the processes of enacting our ‘selves’ is an iterative one that responds to changes in the world. Dings’ (2019) account suggests that behaviour that is incongruent with our sense of self invites reinterpreting or altering the narrative we are guided by. Under the mind-shaping view, to be ‘not myself’ in a given situation would be to act in a way incongruent with the habits I’ve picked up or established in that situation, much like breaking the ‘rules’ of a social situation. To take the bookshop example again, if I am a regular customer who comes to be known as polite and friendly but one day I am rude and dismissive to the book seller, I am – under the mind-shaping view – breaking certain norms of folk-psychological categories that have been applied to me in the past. However, breaking norms invites further interpretation from ourselves and others to explain the incongruity (e.g. “the customer is having a bad day”).

Dings and I importantly differ, however, in our emphasis of the role of the external environment, particularly the social environment, on the interpretative process. Dings (2019) draws on Gibson’s (2014) notion of affordances to describe the ways in which the environment solicits and suggests possibilities for action that are relevant to our personal goals or invite reinterpretation of our goals. Sometimes, however, the environment can both determine what goals are available (like buying books in a bookshop) *and* present challenges to those goals. It is only possible for me, for example, to buy a book from another person, and this can itself be a challenge because the thoughts and experiences of other people are inaccessible to me directly. Other people will be a regular obstacle in the environment to achieving goals in this way. Mind-shaping is a theory that attempts to account for how we are able to overcome that barrier and suggests that, given the prevalence of other people in our environment, working with people is more than a feature of life; it is a *necessity* to realise our goals as people. My account, therefore, incorporates a strong imperative to overcome the obstacles of social understanding. On Dings’ account, obstacles may invite the person in question to reevaluate their goals and change tactics, but my account suggests that social coordination is one such goal we simply can’t get around.

This is not to say that Dings and I are diametrically opposed, but I would add to his account that the social imperative to coordinate with others is a necessary structuring factor to what affordances are available to the agent in question. In this way, I derive from the mind-shaping view that social coordination is an underlying goal to much of our social activity and has a strong influence on what the environment affords.

The relevance and importance of this external imposition to coordinate on our sense of self will become apparent in §5 when I spell out the consequences for self-illness ambiguity. It suffices to say here that individuals who experience mental disorder will also feel the same pressure to socially coordinate. However, I suggest that there is something importantly *transformative* about many mental disorder experiences that can make coordination difficult.

## Mental disorder, the self and transformation

To clarify the key take-away from §3, I want to claim that we are *expected* to enact particular selves in particular situations with some, albeit limited, flexibility in order to facilitate coordination with others. It is also possible that some individuals escape imperatives to coordinate with others, or escape the negative consequences of it, but these aren’t the cases I’m interested in here. I am also not saying that all that there is to the self is the set of norms we conform to; I am merely interested in the implication from mind-shaping that what we call the self has an importantly normative dimension. As such, we should question critically (as I do in §5) what constitutes coordinating well from the mind-shaping perspective and who this might exclude. My thesis is that this excludes individuals with self-illness ambiguities. For the rest of this section, however, I will describe how disorder experiences and the expectations from mind-shaping can come into tension.

The kinds of mental disorder experiences implicated in self-illness ambiguity that I have described (such as eating disorders, depression and anxiety etc.) have been distinctly *transformative* in nature. They involve a shift in a person’s perspective on the world that is either radical or subtle (for example, when someone has started to experience delusions where this was not the case before), which the individual might be more or less aware of, and might be attributed to, or described as, mental disorder. This transformation is a shift in the *mode* of experience; our own experiences are expressed differently, felt differently or acted on differently. For example, considered another way, when a participant in Hope et al.’s (2011) study is asked what it would be like if their anorexia disappeared, they say, “It is slightly different. I can’t put my finger on it, it’s not as if I wouldn’t be me, but I’d be me differently, I guess” (p.25). Similar to the way that the participant feels that they would be ‘themselves differently’ without anorexia, I suggest that transformative disorder experiences are when our unique, embodied perspective on the world is felt differently.

This is not to say that all mental disorders involve a transformative experience, nor that all transformative disorder experiences result in self-illness ambiguities. It is plausible one might have a change in perspective and find it completely unambiguous as to whether that change is down to a different experience of self or a disorder experience. I discuss transformational-type disorder experiences because they highlight the involvement of social norms in a way that make the processes of social understanding transparent. This is because these types of disorder experiences involve change; transformative-type mental disorders, due to the way that someone’s perspective is changed globally, present likely scenarios where someone may act contrary to norms applied to them in the past. For instance, it may be difficult to maintain a certain image of yourself, and act on it, if your outlook has changed. With or without accompanying disorder ambiguities, transformative experiences often require new hermeneutical resources to describe. Transformational experiences of mental disorder will therefore entail a shift in an individual’s system of meaning as typical ways of understanding oneself and the world will no longer be adequate (Spencer, 2023). This is also consistent with Sadler’s (2004) phenomenological account of the disorder experiences implicated in self-illness ambiguity, where he describes how disorder experiences saturate a person’s perspective.

Given that self- and other- understanding is reliant on the enacting of norms (as suggested by the mind-shaping view in §3.1), this shift in personal systems of meaning and understanding also imply that mental disorder may also transform norms of the self. As self-norms are habitual and embodied (see §3.2), transformative mental disorders also entail a shift in embodied habits and ways of interacting in the world (more on this below). Therefore, transformative mental disorder involves a shift in our experience and systems of understanding such that we may act in ways that are incongruent with our own expectations and/or the expectations of others. Mental disorder may thus be emblematic of or involve changes in self-experience.

The transformative nature of some disorder experiences will therefore have important *social* consequences. For example, coordination is affected because we respond to and plan according to how we understand ourselves, and other people similarly plan and respond to how they understand us. Therefore, when our behaviour, thoughts and feelings contradict, undermine or subvert the expectations of the ‘selves’ we perform, coordination can be unpredictable. This is especially the case when we, the individuals with disordered experiences, can’t clearly attribute those behaviours, thoughts and feelings to a ‘self’ concept or ‘disorder’ concept in order to explain the contradiction in norms. Self-illness ambiguity *in particular*, then, may compromise the imperative to coordinate because it may not be clear to the individual in question or others around her what norms (i.e. which self-norms or disorder-norms) are in play.

Self-illness ambiguity is importantly different, however, from cases where it is simply unclear what ‘self’ to enact for the appropriate situation, such as when we are in a situation with two groups of people, each having different expectations of us. For example, we may feel a conflict when our work lives and home lives collide such as at work functions where we can bring our families. In such cases there may be a kind of ambiguity as to whether we should act more like our ‘home self’ or ‘work self’. I do not think the phenomenology of self-illness ambiguity is the tension between a kind of ‘disorder self’ and other kinds of selves. Such experiences may be captured by the concept of ‘multiplex selves’; this idea of the self, described by Hardcastle and Flanagan (1999), captures the experience that we often occupy multiple social roles that may sit uneasily or in tension with one another, but are nevertheless unified. One role in a multiplex self need not undermine the norms of another role; some roles just might be more appropriate than others in a situation, creating a mismatch rather than a contradiction.

Self-illness ambiguities, however, involve experiences of mental disorder that contradict, question, or undermine the norms of the particular self that one is enacting. The norms of one role make it difficult, if not (socially) impossible, to enact the norms of another role. To take the example of anorexia-ambiguities from participant 23’s account (above), if an individual decides not to eat a particular food their behaviour can be understood through the norms of anorexic behaviours (withholding from eating) but it can also be understood through the norms of adult agency (someone is making an informed decision about what to eat). Because eating disorders may be associated with having a lack of agency (something Benschop & Kalis, 2025, challenge), these two interpretations may be incommensurable. I suggest, therefore, that the term self-illness ambiguity specifically refers to instances where a mental disorder experience undermines and contradicts other self-norms that could apply.

Ambiguities involving transformative-type disorder experiences are fruitful examples of this because, in these instances, to paraphrase Hope et al.’s participant (above), we are “ourselves *but different”*. Our habitual way of making sense of the world has importantly changed and therefore it is likely that our old self norms simply cannot meaningfully apply. Nevertheless, we may still be held to the expectations of these old self norms – by ourselves or others – because, for example, it is anticipated that we will still do the same hobbies, do the same job, have the same body, spend time with the same people etc. In essence, ambiguities can occur because, due to the transformative nature of disorder, we may no longer be able to enact the particular kinds of people we are expected to be (‘kinds’ that other people are familiar with or understand).

To compare, one way that physical disorder may create contradictions with the self is through transforming our bodies themselves (see Carel, 2016). Physical disorder symptoms and disability can, for example, change the very shape of our bodies, how we are able to use them and how we feel, such as when we lose the functionality of parts of our body or have to incorporate new technologies into our body (e.g., blood-sugar monitors, pacemakers etc.) This change to embodiment may then undermine self-norms when we are no longer able to participate in those norms. For example, it may be difficult to maintain the image of oneself as a creative person if chronic fatigue interferes with our habitual creative practices.

Mental disorder experiences will also involve transformations of the body. Insofar as the body is our primary tool, or vehicle, for communication (it is the place where expressive acts occur; Merleau-Ponty, 2014), when an experience disrupts that communicative capacity (i.e. through fundamentally changing what is meaningful to us), it follows that the body may also be disrupted. When we struggle to communicate an experience, it is our bodies that struggle to express our meaning. We may, for example, (literally) grasp at words we can’t find. Even when the experience is transformative in such a way that we can continue to communicate, our bodies will still adapt and change to convey new meanings. This transformation can be troublesome for individuals with mental disorder when it leaves them isolated or alienated from others in their social community with whom they had already established a system of understanding and meaning. Changes of experience entail a change in what is meaningful, which the person with mental disorder may then not share with others in their social milieu. For example, an individual with depression might feel alienated from her family because they cannot understand the feeling of bleakness she is trying to convey.

This account isn’t intended as an alternative to the account of self-illness ambiguity described in the introduction and in §2 but is, instead, consistent with a multimodal picture of the self. Bortolan (2022), Maiese (2022) and myself describe, self-illness ambiguity is a phenomenon that is inherently embodied. Our bodies are not only the origins of all our actions, but it also forms the sensorimotor backdrop of our experience (it informs what we *feel* is possible to do, given the structure of our bodies; Merleau-Ponty, 2014) as well as the emotional backdrop of our experience. However, by incorporating a mind-shaping view of social cognition into the picture, which is not currently considered in the literature, we can appreciate that individuals with mental disorder are likely to face additional social pressures that have not yet been theorized (see §5).

In §3 and the above, however, I might be accused of painting a picture of a social environment that is far more rigid than it actually is. Most individuals are not ‘held to account’ by others, with respect to norms, as strictly as I suggest. One may also note, building on this point, that experience is importantly intersubjective; the experience of other people informs my own experience, and we may even perceive together, as in Sartre’s (2003) ‘look’. Therefore, while mental disorder may transform experience in a fundamental way, it will also transform social relationships in a fundamental way; the way that mental disorder changes our thoughts, feelings and behaviours may invite others to also change their thoughts, feelings and behaviours in response. For example, the outgoing person who develops anxiety may invite their loved ones to reflect and adapt their expectations towards the disordered person. This is, I think, consistent with the folk-psychological spiral Andrews (2015; see §3.1) describes. Self-illness ambiguity certainly does not arise whenever someone develops a mental disorder, and so there is certainly flexibility in terms of how others expect us to be.

I may also be falsely implying, because self-illness ambiguity is something we can also experience when other people aren’t around, that when we are alone (and social coordination isn’t a pressing demand) the imperative to coordinate is rigidly internalised. This may suggest that people, in general, conform to norms more than negotiate them. This is plainly not the case as many individuals also ‘adapt’ the norms of their self to these new experiences to create continuity. However, I think that the demand to socially coordinate, at the very least, acts as a guiding post in the processes of understanding given that understanding other people is a ubiquitous problem that humans must face. Social coordination is certainly malleable, especially for those with disorder experiences – we do have norms and folk-psychological categories that ‘explain’ disordered behaviour when it undermines other self-norms – but cases of transformative mental disorder are at least one instance where coordination can be compromised, precisely because it involves an important shift in perspective.

My intention for the last portion of this paper is to show that individuals with self-illness ambiguity face additional challenges as social agents which are not yet captured in the literature. I suggest that we should consider further how disorder experiences are made ‘coherent’ through the adoption of discourses, and how this process thus confers value to specific ways of explaining and giving account of disorder experiences. I suggest that individuals with self-illness ambiguity are particularly marginalised by this value system and thus are highly alienated in society.

## Self-illness ambiguity and giving an account of oneself

As Dings and Glas (2020; see § 2) note, our behaviour is significantly co-regulated by other people, and my mind-shaping approach would support this. Tekin (2013, 2022), and Dings and Glas (2020), might therefore suggest we should be sceptical about how much control we have over the process of constructing the self. Following these authors’ line of thought, I argue we should also be concerned about the wider influences of social structures which can shape whether one’s self and/or mental disorder experience is taken to be understandable.

In order to make this point, building on Anderson’s (2023) work, I think we should understand social cognition, under mind-shaping, to inherently involve *hermeneutic labour*, for example, in the form of work to negotiate norms for successful coordination. Moreover, I suggest individuals involved in processes of social understanding *value* certain types of norms as part of that labour. In other words, only certain norms – in this case, norms around mental disorder – are seen to contribute to the work of social understanding. These norms are attributed value because they are seen to contribute to successful coordination, and, therefore, reaching particular social goals.

Anderson describes hermeneutic labour as,

“the burdensome activity of a) understanding one’s own feelings, desires, intentions, and motivations, and presenting them in an intelligible fashion to others when deemed appropriate; b) discerning others’ feelings, desires, intentions, and motivations by interpreting their verbal and nonverbal cues, including cases when these are minimally communicative or outright avoidant; and c) comparing and contrasting these multiple sets of feelings, desires, intentions, and motivations for the purposes of conflict resolution” (p.178).

Anderson’s description of hermeneutic labour mirrors many of the features of social cognition, especially under the mind-shaping view. As I describe in §3.1, social agents have a stake in making themselves intelligible to others, but it is also necessary to have an understanding of oneself in order to help determine what one should do in a given social situation. This is something that others should be able to grasp and rely on for coordinated activity over time, which also involves being aware of their goals as well as the folk-psychological norms they subscribe to. Social cognition therefore meets criteria ‘a’ and ‘b’ of Anderson’s description of hermeneutic labour. Intelligibility is always at risk, however, as situations change, and people’s behaviour can misalign with expectations. Therefore, it is necessary to negotiate norms as the social situation unfolds. The mind-shaping view, insofar as it uniquely describes the ‘dance’ of conflict resolution in social situations (i.e. the folk-psychological spiral) also maps onto criteria ‘c’ of hermeneutic labour. Mind-shaping should therefore be seen as performing hermeneutic labour, and one that can be, as Anderson describes, ‘burdensome’.

Simply contributing hermeneutic labour, however, doesn’t mean that all is well for individuals with mental disorder. Chapman (2022) argues, for instance, that the way we talk about mental disorder, Madness, and neurodivergence largely (although not exclusively) falls into two types of discourse which can be socially alienating: my disorder, Madness or neurodivergence is something I *am* versus something I *have*. We can understand these discourses as two possible outcomes of the hermeneutic labour we do to explain our experiences to others. Chapman (2022) notes, however, that these discourses (“I have *x*” and “I am *x*”) map onto two different forms of social exclusion. In the case of “I am *x*” discourses, individuals become alienated as a result of their identity being different from mainstream ‘ways of being’. For example, explaining one’s behaviour by saying “I am anorexic” may alienate oneself from the rest of society where this form of identification doesn’t fit into how most people are.

In the other case, when individuals adopt “I have *x*” discourse, people become alienated from other aspects of one’s self. For example, in order to explain unexpected sadness, an individual might have to share with a concerned loved one that they have depression. In this way, individuals may be expected to clearly separate, or put aside, aspects of their experience or behaviour that don’t conform to or undermine the normative expectations of the social situation. Someone might adopt “I have *x*” type discourses as a way to ‘maintain’ or protect some self-norms from disorder norms in order to maintain some form of ‘grip’ on the social coordination task. However, the self may then be alienated from those aspects of experience and behaviour that are less socially ‘useful’ in the situation (i.e. less likely to lead to coordinated success). When carrying out domestic projects in a relationship, which can be difficult when one has depression, an individual may try to separate less ‘useful’ feelings in order to carry out these projects. The consequence of this, however, is that the individual’s experiences of depression are not treated as related to or relevant to their relationship ‘self’.

This analysis of discourses around mental disorder merely highlights a trend that our ways of talking about mental disorder can fall into. Many other variations of these discourses, however, are in use and may be used alongside one another. Regardless of the ways these discourses are used and combined, Chapman (2022) would nevertheless argue that those with mental disorder get stuck in the “neuronormative double bind”, whereby individuals have to choose between adopting a perspective on their disorder experiences that is ‘right’ or ‘useful’ for participating with others, or otherwise position oneself as outside the domain of mainstream participation. It is, therefore, not sufficient to simply state ‘who you are’, in relation to your mental disorder, but you are also expected to give an account of yourself in a way that other people broadly understand. In so far as success is a sliding scale – because the situation you are in constantly shifts – you are likely to be *evaluated* with respect to how well people can coordinate with you. Certain discourses on mental disorder, then, might be valued more than others based on how well other people are able to understand them.

Being understandable in general will be something highly valued as it is likely to lead to successful social interactions, and, because hermeneutic labour is burdensome, individuals are also likely to rely on folk-psychological tools that are familiar. Put simply, sticking with mainstream ways of understanding one’s identity and disorder may be the easiest (and even the best) system for some to get along. Indeed, many individuals might find that these discourses facilitate understanding, empathy and compassion in many circumstances. This is a benefit we should be mindful of.

Nevertheless, as Anderson (2023) describes in the case of gender, these processes of understanding often result in effort distributed unevenly across the parties involved. Being minoritized and marginalised, those with mental disorder arguably have more to lose in a social interaction and thus may be expected to commit more effort to being understandable. There is, therefore, a heavy social burden upon these individuals to be socially understandable in a way that is considered valuable, particularly those ways considered valuable by those with more social power. This chimes with Chapman’s (2023) suggestion that certain types of cognitive activity are valued more than others in society.

Given this, we should be concerned about whether individuals with self-illness ambiguity may lose out on the benefits of social and self-understanding because mental disorder discourses in general may fit self-illness ambiguity experiences poorly. This means that people with self-illness ambiguity will be excluded from the kinds of understanding that discourses around mental disorder and identity can provide. For example, someone with self-illness ambiguity involving an eating disorder may feel alienated from the wider community of individuals with eating disorders where people are able to make use of an identity discourse to demarcate their ‘self’ and their ‘disorder’ (e.g. “That’s my anorexia, not me”). This alienation may be exacerbated because other people may find only *particular* identity discourses understandable, while self-illness ambiguity itself is *not* considered understandable by the mainstream.

This is because, as I characterise them, transformative mental disorders caught up in self-ambiguities can contradict, compromise or undermine the self that someone is trying to present (see §4). Widely used mental disorder discourses may not be an appropriate fit as they construe disorder as continuous with the self in some way (as either integrated with the self or running alongside it). These discourses seem to explain *away* contradiction to make disorder experiences compatible with an idea of the self that oneself and other people can grasp. If one’s experience is *of* a contradiction (as in self-illness ambiguity), these discourses don’t necessarily make those experiences more intelligible. Self-illness ambiguity may then isolate individuals from those whose disorder doesn’t present tensions with the self, and where their experience lies outside typical ways of conceptualising the relationship between self and disorder. People with self-illness ambiguity, then, may not just be alienated from wider society when they cannot conform to norms of mental disorder discourses, but they may also be alienated from mental disorder communities themselves with whom, theoretically, they could share much in common.

In this way, individuals with self-illness ambiguity may be acutely burdened with hermeneutic labour to make themselves understandable by others because their experiences also lie outside the norms for talking about mental disorder more generally. This may also entail that self-illness ambiguities are largely not understandable by people that don’t experience them. Moreover, ways of talking about self-illness ambiguity, as developed by those with lived experience of these ambiguities, may be considered less valuable ways of talking about disorder experience. This would constitute a hermeneutic injustice (see Fricker, 2007) where individuals with self-illness ambiguity:

A) are unable to access appropriate conceptual tools (e.g. discourses) due to poor ‘fit’ with their experience,
B) are limited in their ability to contribute their own conceptual tools (as this may require introducing new norms that others aren’t familiar with, thus limiting how well other people understand),
C) have their conceptual tools devalued (because their experiences may involve inherent inconsistencies), and,
D) are overburdened with expectations of performing hermeneutic labour to conform to more accepted, or valued, normative frameworks of mental disorder and the self.

To conclude, adopting a mind-shaping view of self, as outlined in §3, highlights the ways in which larger social structures, derived from more basic mechanisms of social cognition, are implicated in self and disorder understanding. By interpreting the processes of social understanding as involving labour, namely hermeneutic labour, which is valued differently by different people, we see that *particular* burdens may be placed on individuals with self-illness ambiguity compared to others with mental disorder that have not been highlighted in the literature (see §2). Researchers, going forward, should consider more closely and critically the role of social cognition in phenomena like self-illness ambiguity and how its mechanisms exacerbate the problems that people with self-illness ambiguities can face. In so doing, we may open up new avenues of discourse so that individuals can live these ambiguities unproblematically.

## Conclusion

In §2, I outlined the literature on self-illness ambiguity before unpacking the mind-shaping view in §3.1. In §3.2, I developed the mind-shaping view into an embodied and habitual account of the self, differentiating it from Dings’ (2019, 2020) view through its emphasis on the social expectation to conform to norms. In §4, I described how transformative disorder experiences can undermine self-norms in order to generate inconsistencies which compromise social understanding. Following this, in §5, I draw on Anderson’s (2023) and Chapman’s (2022, 2023) work to argue that certain ways of explaining and accounting for oneself are valued over others, and giving explanations of oneself involves burdensome hermeneutic labour. Individuals with disorder ambiguities are particularly prone to what Chapman (2022) refers to as the ‘neuronormative double bind’ where individuals are expected to characterise their disorder in such a way as to be ‘useful’ or, under mind-shaping, successful for coordination. However, while some people may still benefit from disorder discourse, even if it is alienating, I argued that individuals with self-illness ambiguity are excluded from such benefits because of the experience of incongruities between self and illness that such discourses cannot capture. This leads to a particular form of hermeneutic injustice.

Given the importance that other people play in helping us achieve our personal goals, I would suggest that this acute alienation of individuals with self-illness ambiguity is likely to be detrimental to their sense of agency and possibly even their wellbeing. This implies a need to re-evaluate wider societal practices regarding the inclusion of people with mental disorder, with particular focus on those that can’t clearly demarcate their self from their disorder experiences.

In essence, the mind-shaping view teaches us that the expectations placed on us do not always have our best interests at heart; norms are instrumental and pragmatic for doing things in the social realm. This means that a normatively shaped sense of ‘self’ may have been informed by expectations placed upon a person which are not in their best interest or are not conducive to wellbeing. While I suspect the folk-psychological dance we do to understand others and ourselves in general is rather more of a struggle than a smooth spiral towards our goal, individuals with self-illness ambiguity may face unique problems in being understood.

For one, the normative structures of our social environment may unduly place responsibility on the individual with mental disorder to do the work of ‘fitting in’ in order to live well, for which they may not have the adequate tools or resources. The individual may also simply not want to conform to the standards of understandability dictated by the wider social environment, or by mental disorder communities, and yet feel that they *must*. This could therefore impact one’s personal sense of agency by imposing what shape their ‘self’ should take. By not taking into consideration the wider context of mental disorder discourse or underlying processes of social understanding, we might then fail to make accountable those social structures which reinforce and exacerbate feelings of distress that can come with experiences of self-illness ambiguity.

## Data Availability

N/A.

## Footnotes

1 That’s not to say that there are no self-illness ambiguities for individuals with physical illnesses (§4 would imply this can more definitely be the case, as in my example of creativity and chronic fatigue), but Sadler (2004) thinks, at least, there is something about mental disorder in particular that makes individuals more prone to ambiguity experiences. I do not wholly agree, but do not have space here to defend my point. However, the example above suffices to simply clarify how experiences of disorder and the self might get yoked together.

2 See, for example, Sadler, 2007, and Dings and Glas (2020) on suggestions that the ambiguity should be resolved, and de Haan (2023), Drożdżowicz (2023), McConnell and Golova (2023) on alternative approaches to self-illness ambiguity that don’t rely on disambiguating ‘self’ from ‘disorder’.

## Code availability

N/A.
